# Recommender Systems in Health Professions Education: Protocol for a Scoping Review

**DOI:** 10.2196/69979

**Published:** 2025-08-21

**Authors:** Padraig Mark Healy, Colm O'Tuathaigh, Ciara Heavin, Nora McCarthy, Syed Latifi

**Affiliations:** 1 School of Medicine Medical Education Unit University College Cork Cork Ireland; 2 Division of Medical Education Weill Cornell Medicine – Qatar Doha Qatar; 3 Cork University Business School University College Cork Cork Ireland

**Keywords:** recommender systems, precision education, personalized learning, health professions education, HPE

## Abstract

**Background:**

In health professions education (HPE), the concept of precision education is being explored, with the intention of tailoring learning experiences to the unique needs of learners. Recommender systems can assist academic decision-making. They can be used to personalize content delivery, suggest appropriate learning pathways, propose schedules, recommend suitable institutes, supervisors, and courses, and provide learner feedback. Given abundant learning resources, selecting the right one can be daunting. Recommender systems may address this challenge by offering tailored suggestions that align with learners’ requirements and abilities.

**Objective:**

This study aims to examine the literature related to the use of recommender systems in HPE.

**Methods:**

This review will be conducted following the methodological framework proposed by Arksey and O’Malley. A comprehensive search will be conducted across the MEDLINE, CINAHL Plus with Full Text, ERIC, Academic Search Premier, and Web of Science databases, as well as gray literature sources including arXiv and Google Scholar. These searches will focus on the period from January 2000 to February 2025. In addition, backward and forward citation searching will be carried out. Articles will be screened independently by 2 reviewers; discrepancies resolved by consensus or a third reviewer. The selection process will involve an initial screening of titles and abstracts to identify potentially relevant articles. If initial screening is inconclusive, full-text review will ensure articles meet inclusion criteria. The main eligibility criteria for inclusion in the review are studies involving health professions students or educators, focusing on the concept, development, or application of recommender systems. Data extraction will be performed using a customized data charting template covering article, study, and recommender system details. The extracted data will be analyzed and displayed in both tabular and graphical formats, supplemented by a narrative interpretation. The findings will be synthesized by mapping the existing literature to identify key concepts, research gaps, and types of evidence, highlighting similarities and differences in how recommender systems are applied in HPE. This reporting will be in accordance with the PRISMA-ScR (Preferred Reporting Items for Systematic Reviews and Meta-Analyses Extension for Scoping Reviews) guidelines. Data extraction and analysis will be conducted using Covidence.

**Results:**

The current phase of the study involves selecting studies for the scoping review as specified in this protocol. The search, screening, and data extraction will begin in February 2025. The results of the study and the submission of a manuscript for peer review are expected in the winter of 2025.

**Conclusions:**

This study aims to comprehensively map the extent of recommender systems in HPE. By identifying effective practices and existing gaps, it will serve as a valuable resource for health professions educators, enabling them to make informed decisions about integrating these systems into educational applications.

**International Registered Report Identifier (IRRID):**

PRR1-10.2196/69979

## Introduction

### Overview

The magnitude of medical information and learning resources available online, while advantageous to the user, can also be overwhelming [1-4]. The sheer volume and varying quality can lead to information overload [5-7]. This can be a stressor, making it difficult for one to evaluate the relevance and quality of the information available, ultimately impacting the individual and inhibiting effective decision-making [8-10]. In addition, there are concerns about the credibility and validity of medical and health-related information found on widely used information-sharing platforms [11,12]. A systematic review by Wang et al [13] showed an increasing trend in health-related misinformation on topics such as vaccination, Ebola, and Zika virus. Coincidentally, the article was published a month before the COVID-19 pandemic started making headlines, which itself garnered attention from online “misinformation superspreaders” [14]. Furthermore, YouTube has been identified as a source of misinformation regarding the human papillomavirus vaccine [15]. Studies have also highlighted the spread of cancer misinformation on Facebook [16,17], misinformation related to e-cigarettes and nicotine on Twitter [18], and misinformation about rheumatoid arthritis across various platforms [19]. These issues are problematic because they can lead to public health risks, such as vaccine hesitancy [20], poor disease management [21], and the spread of harmful health practices [22].

In response to these challenges of information overload and the accuracy of information, recommender systems are emerging as a powerful tool [23-25]. Recommender systems, also known as recommendation systems, platforms, engines, or algorithms, are essential tools in information filtering and personalization [26,27]. Their use can improve user experiences, boost engagement, and aid decision-making [28]. A seminal article by Resnick and Varian [29] characterized a recommender system as one in which recommendations are provided as inputs by people, which the system then aggregates and directs to appropriate recipients. Burke [30] described them as systems that generate tailored recommendations or assist users in a personalized way to choose from an array of options. These systems use algorithms to tailor recommendations by filtering, curating content, and guiding the user toward pertinent resources [31,32]. Thus, recommender systems play a pivotal role in alleviating the burden of information overload.

Recommender systems have already seamlessly integrated into our everyday lives. Serving as intelligent guides, they suggest relevant content to users in contexts ranging from social media to e-commerce and streaming services. According to a report by MacKenzie et al [33], 35% of Amazon purchases and 75% of Netflix viewing choices can be attributed to recommendations by such algorithms. In recent years, approaches such as machine learning, natural language processing, and deep learning [34-37] have brought advancements to recommender systems. These systems are not only prevalent in commercial applications but are also increasingly being adopted in educational settings [38,39]. A recent systematic review by Thongchotchat et al [40] found that while attribute- or characteristic-type recommender systems dominate education, there is an emerging trend of varied systems, including hints, clues, prompts, and tutoring, but these are in the early stages and require exploration and development. Combining theories of learning styles may enhance the flexibility and effectiveness of recommendations [40]. A recent bibliometric analysis [39] noted an increase in research dedicated to exploring recommendation techniques with the objective of enhancing the accuracy of recommendations made by educational recommender systems, driven by the ever-increasing amount of available learning resources. Large language models (LLMs) now enhance conversational recommender systems by leveraging their contextual understanding, potentially improving recommendation quality [41,42]. Recommender systems fall under 1 of 4 types of filtering approaches [43-45]. Table 1 provides a detailed description of each type, including its respective strengths and limitations, as well as examples.

**Table 1:** Comparison of recommender system filtering types.

Filtering type	Description	Strengths	Limitations	Examples
Content	A recommendation is made based on user characteristics or past behavior (ie, a user’s data) [46].	Gray sheep: adapts to users with unique profiles [47]. Recommendations can be shared without the user sharing a profile, thereby ensuring privacy [48]. Can recommend new, rare, or unpopular items [49].	Lack of serendipity: may not suggest unexpected items, as it does not rely on data from other users [50]. New user problem: difficult to make recommendations in the absence of historical data to understand the user [51].	A study app that recommends quiz questions similar to those the user is practicing.
Collaborative	A recommendation is made by identifying patterns among similar users [52].	Leverages the power of the collective user behavior [53]. Can recommend items that a user may not have found independently [54].	Cold start problem: difficult to make recommendations with little or no historical data [55]. Sparsity: many user-item interactions may be unknown [56]. May only recommend popular items [57]. Raises privacy concerns, as data must be shared [27]. Gray sheep: Not effective for users with unique profiles [58].	A clinical skills training app that recommends practice scenarios frequently used by other students.
Knowledge	A recommendation is made based on explicit domain user knowledge and recommendation criteria [59].	Useful when there is explicit knowledge about an item or user preferences [60]. New item problem: can be managed effectively, as it does not rely on past user-item interactions [61].	Knowledge acquisition bottleneck: requires explicit knowledge, which may not always be obtainable [62]. Lack of serendipity: may not suggest unexpected items, as it relies on the user’s requirements rather than on data from other users [63].	An advising system that recommends research opportunities and projects based on a student’s academic background and research interests.
Hybrid filtering	This approach uses ≥2 filtering techniques [30].	Combines techniques to overcome the limitations of a single approach [64].	Sensitive to the strengths and weaknesses of the techniques used [65].	A study platform that, for learners of a specific module, may adopt a hybrid approach— (1) content-based: recommend additional resources similar to those used in the module, and (2) collaborative-based: recommend resources that other learners studying the same module found helpful.

### Recent Advances in Artificial Intelligence and LLMs in Recommender Systems

The integration of generative artificial intelligence and LLMs has advanced the development of recommender systems. These technologies enhance natural language processing, facilitate natural-sounding dialogues, and improve recommendation accuracy and user interaction experiences [66-68]. Approaches such as retrieval-augmented generation models combine retrieval models and generative models to integrate specific knowledge sources, aiding complex recommendations while also addressing issues such as hallucinations and cold starts [69,70]. These advances, however, are not just restricted to text-based systems. Multimodal recommender systems are emerging in popularity, integrating various types of input data such as text, image, and audio. By leveraging the capabilities of LLMs to process this multimodal data, more comprehensive and accurate recommendations are possible [71,72]. Despite these advancements, challenges remain. Biases in LLM-based recommendations [73] and ethical concerns continue to pose challenges [74]. As these systems continue to evolve, they will offer more efficient and personalized solutions.

### Merits and Use Cases in Health Professions Education

Learners in health care seek reliable materials for their education online [75,76], while others turn to the internet to understand their health conditions, treatment options, and preventive measures [77-81]. YouTube, a popular educational resource among health profession students [82,83], raises concerns for health care educators regarding the quality of information presented to students. This concern is supported by findings from Helming et al [84], who found that many YouTube videos on medical education are of low quality, even those created by academic physicians. Metrics such as views and likes, while popular among learners for navigating their choices, can be superficial and unreliable indicators of quality or accuracy [11,83,85]. Similarly, a study by Camm et al [86] found no correlation between video quality as assessed by a scoring tool and commonly used preference metrics (ie, hits, likes, dislikes, or search page rankings). When it comes to the videos recommended by the platform, the rationale behind the algorithm for suggesting specific content remains unclear? By developing a proprietary recommender system curated by experts in the subject matter, the suggested information and learning resources could be moderated, ensuring the quality and relevance of the materials recommended to learners.

Educational recommender systems are information systems designed for use in educational settings to recommend various resources to different stakeholders, including learners, educators, researchers, and others [87]. Leveraging advanced algorithms and data analytics, educational recommender systems provide tailored recommendations that enhance the educational experience by addressing individual learner needs and preferences [1,38]. They facilitate efficient resource discovery, aiding personalized learning and informed decision-making in academic applications [38,40]. These systems are used in a variety of ways, with the goal of assisting students with academic decisions. They are used to recommend suitable institutes, study topics, and courses [88-90]. They can be used to propose personalized syllabi and scheduling [91-93], learning materials [94-97], scholarships [98], supervisors [99], and career paths [100]. These systems can also be used to offer learners feedback on summative assessments [101]. In education, personalized learning centers on learners, acknowledging their inherent differences—variations in strengths, weaknesses, and learning preferences [102]. This recognition of differences suggests that a uniform or “one-size-fits-all” approach may not be optimal for all learners. Koestner et al [103] conducted a meta-analysis that revealed that learners are more likely to achieve their goals when those goals are (1) self-concordant and (2) accompanied by an implementation plan. The concept of agency, as defined by Bandura [104], refers to the capacity to initiate actions with intention and purpose. In education, this concept manifests as learner agency—a framework that embodies self-directed learning [105,106]. Learners actively pursue knowledge aligned with personal goals and interests, facilitated by strategic guidance from educators [107,108]. Educators play a crucial role in this process by providing the necessary guidance and support to ensure that learners have access to high-quality and relevant resources. In addition to the information overload highlighted earlier, learners may sometimes be unaware of what to search for [109]. Deschênes [110] highlighted that recommender systems are valuable for curating educational resources tailored to the needs of individual learners, empowering informed choices, guiding learners to resources (that they may not have encountered unprompted), and fostering an environment conducive to autonomous yet guided education.

While the topic of recommender systems has been extensively studied in both health care [111,112] and education [113-115], to the best of the authors’ knowledge, there appears to be a gap in the literature regarding their application in health professions education (HPE). For example, a topic search (covering the title, abstract, and keywords) on Web of Science using the terms “recommender system*” and “health professions education” returned only 2 articles. This scoping review aims to comprehensively map the extent of recommender systems in the HPE domain.

### Practical Implementations in HPE

In reviewing the literature for applications of such systems in HPE, several innovative implementations with diverse purposes have been identified. One system provides adaptive, competence-based recommendations tailored to each student’s unique needs [116]. Another is used to evaluate nursing competencies and to recommend targeted learning materials to address skill gaps [117]. Liou and Chen [118] presented an e-learning platform that recommended personalized learning activities for medical interns. Another system assists second-language nurses in patient care charting by suggesting optimal terms during documentation [119]. Liou [120] introduced a recommender system for an online discussion forum that personalized article recommendations based on individual preferences.

### Objectives

This scoping review aims to identify the different use cases of recommender systems in HPE. In addition, this review aims to determine the filtering techniques used by these systems, analyze their attributes or features, and explore the user experiences of both learners and educators. Furthermore, this review seeks to identify gaps in the current literature and provide suggestions for future research on the use of recommender systems in HPE.

## Methods

### Ethical Considerations

This study is based on the analysis of published literature, both scientific and gray. It does not involve patients, medical research, or any type of personal information, and, as such, no ethics approval is required. The results of this scoping review will be submitted for publication in a peer-reviewed international journal and presented at scientific meetings and conferences related to HPE.

### Protocol Design

#### Overview

Scoping reviews have gained popularity in recent years as a research methodology [121]. The objective of a scoping review is to identify and map the evidence available on a given topic [122]. Munn et al [123] elaborated on the objectives of conducting a scoping review by outlining six distinct components: (1) catalog the breadth of evidence available, (2) clarify concepts or definitions, (3) examine how the available research is conducted, (4) identify key characteristics of the topic, (5) ascertain the feasibility of conducting a systematic review, and (6) identify gaps in the existing literature. The methodology used in this scoping review is based on that of Arksey and O’Malley [122]. To ensure rigor, quality, and reproducibility, a recommended checklist [124,125] will be followed: the PRISMA-ScR (Preferred Reporting Items for Systematic Reviews and Meta-Analyses Extension for Scoping Reviews) [126]. The output will be displayed using a flow diagram.

#### Stage 1: Identifying the Research Question

The objective of this scoping review is to gain a better understanding of the current use of recommender systems in HPE. Specifically, we will investigate the following questions:

1. How are recommender systems being applied in HPE? That is, what is the recommendation element?
2. What filtering technique or techniques are used by recommender systems in HPE?
3. What are the attributes or features of these HPE recommender systems?
4. What are the experiences of users, that is, learners and educators in the health professions, regarding the use of recommender systems?
5. What are the gaps in the current research on the use of recommender systems in HPE?

#### Stage 2: Identifying Relevant Studies

A literature search will be conducted across the selected databases, consisting of the medical-specific source (MEDLINE), the nursing and allied health database (CINAHL Plus with Full Text), the educational search engine (Education Resources Information Center), and the multidisciplinary databases (Academic Search Premier and Web of Science). Gray literature will be searched via the arXiv repository for preprints and Google Scholar. For gray literature sources, we will review the first 100 results, ranked by the platform’s relevancy sorting feature. For Google Scholar, the searches will be limited to article titles, an approach recommended by Haddaway et al [127] for conducting systematic reviews. This comprehensive approach will ensure a high-quality literature review by retrieving a wide range of potentially relevant articles from a broad multidisciplinary perspective.

A search-string development strategy will be developed in accordance with the 3-step recommendations provided by the Joanna Briggs Institute [128]. First, an initial limited search will be conducted in 3 databases: Academic Search Premier, CINAHL, and MEDLINE, with the relevance of the terms used being evaluated. Next, the text in the returned articles (titles, abstracts, and keywords) will be analyzed. A comprehensive search will then be performed using these terms across the databases. Finally, citations in the retrieved results will be manually reviewed to identify any additional relevant studies not captured in the database or gray literature searches.

The search strategy will include title and abstract searches with terms related to HPE and recommender systems, connected using Boolean operators (AND, OR, and NOT). Controlled vocabulary terms (eg, Medical Subject Headings [MeSH] terms, subject headings, or subject terms) will be used when available in the database to enhance the search, independent of the title and abstract search parameters. These terms will help identify synonyms and related concepts, thereby broadening the search scope. Truncations (or stemming) will be used to capture the root of words, ensuring that different endings of the term of interest are included. For example, using “recommend* system” would capture “recommendation system,” “recommender system,” and “recommending system.” In addition, we will implement word proximity searches to ensure that terms of interest are situated within a specified number of words from each other. This approach will allow us to capture relevant phrases and contexts that are not caught through truncation alone. For example, when we search for “recommender N2 system*,” articles in which “recommender” appears within 2 words of any variation of “system” will be returned. For instance, it would capture a sentence such as, “The recommender model system enhanced student learning outcomes,” which would otherwise be missed.

The following are examples:

1. TI = (recommender NEAR/2 system* OR recommendation NEAR/2 system*) OR AB = (recommender NEAR/2 system* OR recommendation NEAR/2 system*)
2. TI = (medical OR nurs*) OR AB = (medical OR nurs*)
3. 1 AND 2

This example includes a title and abstract search, making use of Boolean operators (AND and OR), stemming (eg, recommend*), and proximity search (eg, NEAR/2).

The search period will span from January 2000 to February 2025. Our decision to start the scoping review in 2000 is informed by existing literature on recommender systems in education. The systematic review by Urdaneta-Ponte et al [129] focused on literature from 2015 onward, while the work by Kamal et al [89] covered 2011 onward. The literature review by Lampropoulos [114] traced studies on the use of recommender systems in education back to 2001. By selecting the earliest date from these studies, we establish a conservative starting point, minimizing the risk of overlooking relevant literature. The search strategy targets students and educators in specific health professions, including medicine, nursing, dentistry, pharmacy, public health, allied health, clinical education, veterinary medicine, paramedical sciences, occupational therapy, physiotherapy, speech therapy, audiology, mental health, biomedical sciences, nutrition, dietetics, midwifery, chiropractic, and podiatry. The inclusion criteria will be limited to studies reporting on the concept, development, or application of recommender systems applied to diverse educational or training offerings designed for these health professions students or educators (eg, undergraduate, postgraduate, continuing professional development). Studies involving mixed populations, that is, health professions and non–health professions students, will be excluded. For a detailed view of how search lines 1 and 2 in the example above are combined, a sample search strategy can be found in Multimedia Appendix 1.

All identified references will be imported into the reference management software, EndNote [130], to merge and manage the citations and remove any duplicate entries. Once the data cleansing is complete, the citations will be imported to Covidence [131] for the study selection screening and charting stages.

#### Stage 3: Study Selection

Next, a 2-stage screening process will be implemented. The first stage involves scanning titles and abstracts to identify relevant articles. To be eligible for the second stage, the title or the abstract (or both) must (1) focus on the use of recommender systems and (2) target HPE. The second stage involves a full-text review of articles. In both stages, reviewers will screen articles against the eligibility criteria outlined in Textbox 1. Any uncertainty about whether an article should be included during the initial screening will result in its progression to the full-text review stage. Disagreements about the relevance of an article during the full-text review will be discussed among the reviewers, and if no consensus is reached, a third reviewer will be consulted to decide whether to include the article. The reasons for excluding the articles will be documented in the scoping review report.

**Full inclusion and exclusion criteria.**

**Inclusion criteria**

- Population: studies involving health profession students or educators (including undergraduate, postgraduate, and continuing professional development [CPD]).
- Concept: studies focused on the concept, development, or application of recommender systems.
- Context: any geographic location
- Types of evidence: all primary studies of all designs; systematic reviews; full-text conference proceedings; full-text articles; articles published from 2000 to February 2025
- Language: any

**Exclusion criteria**

- Population: studies that exclude health profession students or educators (eg, those centered on patients or the public); studies with mixed populations (ie, health professions and non–health professions students)
- Concept: studies that do not address recommender systems (eg, studies focused on unrelated technologies)
- Context: none
- Types of evidence: articles published before 2000; meeting abstracts

#### Stage 4: Charting the Data

Data extraction will be conducted by 2 independent reviewers (reviewers 1 and 2) to ensure consistency and reliability. A standardized data extraction form, developed by the research team and configured in Microsoft Excel, will be used to chart the relevant data from all included studies (Table 2). The extracted data will cover 4 domains: article details, study details, specifics of the recommender system, and user experience. Each domain will be carefully examined to ensure that the collected data aligns with the research questions, and discrepancies will be noted for further analysis.

For article details, information such as article type (eg, original research, review, and case study), authors’ names, affiliations or institutions, year of publication, and the country of the study’s origin will be included to help contextualize the study within the broader literature. The study details will cover the study design (eg, observational, longitudinal, randomized controlled trial, and qualitative), the primary objective of the study, participant demographics, and the main outcomes of the study, providing insights into methodologies and key findings. In addition, details about the recommender system will be documented, including its application (ie, what it is recommending), the filtering techniques used, the attributes or features of the recommender system, and the user experiences of learners and educators, revealing practical applications and features, as well as highlighting user feedback. Data charting will be conducted using Covidence, a cloud-based resource for scoping review management.

This form will be piloted on a subset of articles to assess its effectiveness. For instance, certain important characteristics of the articles may not be captured by the current items, requiring adjustments to the instrument. The full-text reviewers will be asked during the pilot stage to identify any additional variables that should be considered for charting, and the form will be revised if deemed necessary. Any disagreements between the reviewers will be resolved through discussion, with a third reviewer (reviewer 3) acting as an arbiter if necessary.

**Table 2:** Data charting instrument.

Domain and subdomain		Description or examples
**Article**		
	Article type	Original research, review, and case study
	Authors	List of authors’ names
	Affiliation	List of authors’ affiliations or institutions
	Year of publication	Year the article was published
	Country	Country of the study’s origin
	Limitations	Any limitations described or observed in the article
**Study**		
	Study design	For example, observational, longitudinal, randomized controlled trial, and qualitative
	Aim	Primary objective or purpose of the study
	Participants	Who were the participants in the study?
	Outcomes	Main results of the study
**Recommender system**		
	Element	How is the recommender system being applied? That is, what is it recommending?
	Technique or techniques	What filtering technique or techniques are used by the recommender system?
	Attributes	What are the attributes or features of these health profession education recommender systems?
	User experience or experiences	What are the experiences of users (ie, learners and educators) in the health professions regarding the use of recommender systems?

#### Stage 5: Collating, Summarizing, and Reporting the Results

Data will be analyzed and summarized, with the study characteristics presented in both tabular and graphical formats, accompanied by a narrative interpretation. To ensure thorough synthesis, we will use a framework that categorizes the data according to our research questions. This will involve mapping the extracted data to each question, identifying patterns, and summarizing the aggregated evidence. For example, we will compare the filtering techniques (eg, content, collaborative, knowledge, or hybrid) used across different studies to understand their application in various HPE contexts. We will create summary tables that outline the key findings from the included studies for each research question. This approach will help us systematically present the different use cases and characteristics. In reporting our results, we will highlight the similarities and differences in how recommender systems are applied in HPE and identify research gaps through a comparative analysis of the studies and their applications. Arksey and O’Malley [122] suggest an optional sixth stage—stakeholder consultation—to gather feedback on the findings uncovered during the scoping review. Although we acknowledge the value of this step, this scoping review is part of a larger study that will later include a qualitative component involving stakeholders. Therefore, this scoping review will not include a stakeholder consultation.

Table 3 provides an estimated timeline for this scoping review. This timeline is designed to balance the thoroughness required for a comprehensive literature review with the need for timely completion to maintain the relevance of our findings to the academic community.

**Table 3:** Data charting instrument.

Stage	Description	Estimated completion date
1	Identifying the research question	October 2024
2	Identifying relevant studies	February 2025
3	Selecting studies	June 2025
4	Charting the data	September 2025
5	Collating, summarizing, and reporting the results	November 2025

## Results

The study will begin the phase of selecting studies for this review as outlined in the protocol, including the processes of search, screening, and data extraction, in February 2025. We anticipate completing the study and submitting the manuscript for peer review by the winter of 2025. This review aims to provide a detailed overview of the various applications of recommender systems in HPE, including the filtering techniques used and the specific features of these systems.

## Discussion

### Anticipated Findings

The anticipated findings of this scoping review are expected to provide valuable insights into the diverse applications and potential benefits of recommender systems in HPE. These systems can significantly enhance learning experiences by personalizing the content to the unique needs of individual learners. By providing tailored recommendations, these systems can help bridge knowledge gaps, reinforce learning, and support continuous professional development. The expected findings will provide insights into the experiences of both learners and educators, identifying applications and implementation challenges. The scoping review aims to evaluate existing evidence and identify gaps in the literature concerning the use of recommender systems in HPE.

### Comparison to Prior Work

Previous studies have highlighted the benefits of recommender systems in various educational contexts of HPE (116-20). A continuously growing number of learning resources has created a need for these systems in education [23-25,39]. Digel et al [38] reported that engaging users in the development process of such systems is crucial to ensure their acceptance and use. It is anticipated that the applications and implementation challenges identified through this scoping review will help health profession educators in developing future systems. This review will build on these findings by specifically focusing on their use with HPE and identifying unique challenges and opportunities in this field.

### Strengths and Limitations

A strength of this review is its comprehensive methodology, which includes a thorough search across multiple scientific databases and gray literature sources. This approach ensures a broad and inclusive overview of the existing literature. However, some limitations should be acknowledged. First, because the screening processes for scoping review do not assess the quality of the included studies, the robustness of the evidence presented may be impacted [132]. In addition, by focusing exclusively on studies published from 2000 onward, the review may overlook earlier research that could offer valuable insights and increase the potential for systematic bias [133]. Furthermore, while the aim is to cover a broad range of studies related to recommender systems, this breadth may come at the cost of a lack of depth in the analysis [134], making it difficult to draw specific conclusions. Publication bias, in which the likelihood of a study being published is influenced by the nature and direction of its results, creates a systematic difference between published and unpublished studies. Although incorporating gray literature in our study helps mitigate this bias, it does not fully eliminate it, as studies with positive or significant outcomes are still more likely to be published [135].

### Dissemination Plans

The dissemination strategies for the findings of this scoping review will include publication in a peer-reviewed journal and presentations at national and international conferences. The dissemination activities will commence in the winter of 2025 with the manuscript submission.

### Conclusions

While recommender systems have been extensively studied in health care and education, there is a significant gap in the literature regarding their application in HPE. To the best of our knowledge, this is the first comprehensive exploration of recommender systems specifically focused on HPE. This scoping review aims to thoroughly map the extent and various applications of these systems within this domain. By identifying effective practices and highlighting existing gaps, this review will serve as a valuable resource for health professions educators, enriching academic discourse and providing practical guidance for effective implementation in HPE.

## Appendix

Multimedia Appendix 1 Search strategy for the MEDLINE database.

Multimedia Appendix 2 PRISMA-ScR Checklist.

## Abbreviations

HPE: health professions education
LLM: large language model
MeSH: Medical Subject Headings
PRISMA-ScR: Preferred Reporting Items for Systematic Reviews and Meta-Analyses Extension for Scoping Reviews

## Data Availability

Data sharing is not applicable to this paper as no datasets were generated or analyzed during this study.
