# Supplementary material for: Recommender Systems in Health Professions Education: Protocol for a Scoping Review
Source: JMIR Res Protoc. 2025 Aug 21;14:e69979. doi: 10.2196/69979 (PMC12411800; doi:10.2196/69979)
Supplement: Multimedia Appendix 1 [file resprot_v14i1e69979_app1.docx]

**Appendix I: Search strategy for Medline**

| **Query** | **Records retrieved** |
| --- | --- |
| 1 \| ((recommender ADJ2 system*) OR (recommendation ADJ2 system*) OR (recommend* ADJ2 platform*) OR (recommend* ADJ2 engine*) OR (recommend* ADJ2 algorithm*) OR (personal* ADJ2 learning) OR (adaptive ADJ2 learning) OR (collaborative ADJ2 filter*) OR (content-based ADJ2 filter*) OR (knowledge-based ADJ2 filter*) OR (hybrid ADJ2 recommender*)).ti,ab. |  |
| 2 \| (medical OR nurs* OR pharmac* OR healthcare OR "public health" OR "allied health" OR "health sciences" OR "clinical education" OR dental OR veterinar* OR paramedical* OR "occupational therapy" OR physiotherapy OR "physical therapy" OR "speech therapy" OR audiology OR "mental health" OR "health professions" OR biomedical OR nutrition* OR dietetics OR midwifery OR "health training" OR "medical training" OR "clinical training" OR chiropractic OR podiatry OR "health and social care").ti,ab. |  |
| 3 \| exp Education, Dental/ OR exp Education, Medical/ OR exp Education, Nursing/ OR exp Education, Pharmacy/ OR exp Education, Public Health Professional/ OR exp Students, Dental/ OR exp Students, Medical/ OR exp Students, Nursing/ OR exp Students, Pharmacy/ OR exp Students, Public Health/ |  |
| 4 \| ((online ADJ2 education) OR (online ADJ2 learning) OR eLearning OR (electronic ADJ2 learning) OR (virtual ADJ2 learning) OR (distance ADJ2 education) OR (distance ADJ2 learning) OR (blended ADJ2 learning) OR (hybrid ADJ2 learning) OR (remote ADJ2 learning) OR (digital ADJ2 education) OR (digital ADJ2 learning) OR (web-based ADJ2 learning) OR MOOCs OR "Massive Open Online Course*" OR (technology-enhanced ADJ2 learning) OR (internet-based ADJ2 education) OR (virtual ADJ2 classrooms) OR (online ADJ2 course*) OR (asynchronous ADJ2 learning) OR (synchronous ADJ2 learning) OR (computer-assisted ADJ2 learning) OR (cyber ADJ2 education) OR (mobile ADJ2 learning) OR m-learning OR (supervised ADJ2 learning) OR (unsupervised ADJ2 learning)).ti,ab. |  |
| 5 \| 2 OR 3 |  |
| 6 \| 1 AND 4 AND 5 |  |
| 7 \| #6 Limited to 2000 to Current | 123 |

**Parameter(s) applied:**

Publication Date: January 2000 to February 2025

Date: 2^nd^ Feb 2025
